# Differentially Private Client Selection and Resource Allocation in Federated Learning for Medical Applications Using Graph Neural Networks

**DOI:** 10.3390/s24165142

**Published:** 2024-08-08

**Authors:** Sotirios C. Messinis, Nicholas E. Protonotarios, Nikolaos Doulamis

**Affiliations:** 1 Institute of Communication and Computer Systems, National Technical University of Athens, 15773 Athens, Greece; ndoulam@cs.ntua.gr; 2 Mathematics Research Center, Academy of Athens, 11527 Athens, Greece; nprotonotarios@academyofathens.gr

**Keywords:** decentralized federated learning, resource allocation, differential privacy, client selection, graph neural networks

## Abstract

Federated learning (FL) has emerged as a pivotal paradigm for training machine learning models across decentralized devices while maintaining data privacy. In the healthcare domain, FL enables collaborative training among diverse medical devices and institutions, enhancing model robustness and generalizability without compromising patient privacy. In this paper, we propose DPS-GAT, a novel approach integrating graph attention networks (GATs) with differentially private client selection and resource allocation strategies in FL. Our methodology addresses the challenges of data heterogeneity and limited communication resources inherent in medical applications. By employing graph neural networks (GNNs), we effectively capture the relational structures among clients, optimizing the selection process and ensuring efficient resource distribution. Differential privacy mechanisms are incorporated, to safeguard sensitive information throughout the training process. Our extensive experiments, based on the Regensburg pediatric appendicitis open dataset, demonstrated the superiority of our approach, in terms of model accuracy, privacy preservation, and resource efficiency, compared to traditional FL methods. The ability of DPS-GAT to maintain a high and stable number of client selections across various rounds and differential privacy budgets has significant practical implications, indicating that FL systems can achieve strong privacy guarantees without compromising client engagement and model performance. This balance is essential for real-world applications where both privacy and performance are paramount. This study suggests a promising direction for more secure and efficient FL medical applications, which could improve patient care through enhanced predictive models and collaborative data utilization.

## 1. Introduction

Federated learning (FL) is a distributed learning paradigm, in which models are trained across multiple devices without direct transfer of raw data to a centralized server [1]. In this paradigm, training involves collaboration between multiple clients, such as research institutions, hospitals, and medical devices, enhancing privacy among the involved parties [2]. FL components facilitate the scalability of machine learning applications by ensuring the protection of personal data at the edge [3].

In the context of medical devices, FL enables the training of machine learning models by using data from a vast array of devices [4,5]. To this end, each device contributes its data to the training process, allowing the model to learn from a diverse range of patient populations and clinical scenarios [6,7]. Furthermore, FL can aid in tackling issues associated with data heterogeneity, given that medical devices often produce data of varying characteristics and formats. However, the data transfer required by distributed learning algorithms significantly consumes radio resources, including energy and bandwidth, which are frequently limited in real-world scenarios [8]. Bandwidth allocation, a subject of extensive study in the resource allocation problem for FL (see, for example, [9]) is often integrated with various approaches, such as agent selection and power control, to improve FL’s communication efficiency. Simultaneously, achieving satisfactory FL performance under energy limitations is also essential. Moreover, the loss functions of agents depend on their private data, which may include sensitive information, such as health or financial records. Even though direct exchange of raw data is avoided in distributed learning settings, the information shared between agents and servers can be vulnerable to interception by malicious entities, potentially allowing them to infer the agents’ private data [10].

**Figure 1:**
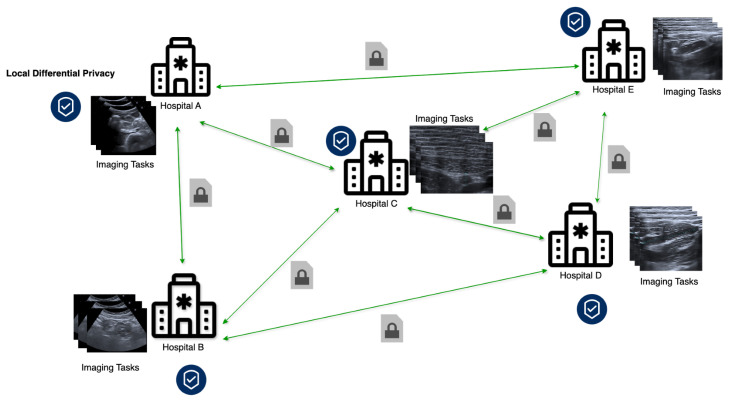
Decentralized federated learning environment in healthcare.

Although traditional FL relies on a centralized server to coordinate model training across distributed devices, there is an increasing need for serverless FL settings, usually referred to as decentralized federated learning (DFL) [11]. DFL operates on the decentralized model exchange between clients, offering resilience against single-point failures and reducing network congestion through direct device-to-device (D2D) communications [12], see Figure 1. Despite these advantages, DFL faces several challenges, including long delays due to frequent decentralized model updates, potentially increasing communication and computation resource usage. Heterogeneity among devices and the decentralized structure may result in stragglers with slower updates, reducing aggregation frequency and impacting convergence rates.

Cross-device FL has been widely applied in diverse fields, including mobile phones, internet of things (IoT) devices, and mobile edge computing. In these settings, DFL client devices exhibit considerable variation in data characteristics and system configurations; inadequately addressing this diversity may affect performance [13]. Therefore, managing client heterogeneity has become a key priority. DFL client selection, also addressed as participant selection or device sampling, plays a pivotal role, as it determines the client devices in each training round. Effective client selection strategies can greatly improve model performance, in terms of accuracy, fairness, robustness, and efficiency by reducing training overheads [14]. Nonetheless, the risk of revealing private information persists through the analysis of uploaded client parameters, such as the weights in deep neural networks [15].

Furthermore, in both FL and DFL, ensuring privacy is critical to the user. This fact has led to the adoption of the differential privacy (DP) technique, which provides a rigorous mathematical framework for quantifying and minimizing the privacy risks associated with sharing sensitive data during the training process [16]. DP is a technique used to protect the privacy of individuals in large datasets by adding random noise to the data, in such a way that statistical analysis of the data remains accurate without revealing the identity of individual data points [10]. It is particularly useful in healthcare and medical devices, where it helps collect, analyze, and safeguard sensitive health and medical information from unauthorized access [17]. Furthermore, DP is essential in FL applications, to protect individual data, build user trust, and comply with privacy regulations [18]. By introducing controlled noise into the learning process, DP enhances protection against attempts to infer membership or re-identify individuals, thereby complicating efforts by adversaries to obtain sensitive information. This privacy-preserving framework ensures balance between model utility and user privacy, thus encouraging broader participation in FL, while future-proofing against evolving privacy concerns [19].

In FL environments, where client connections usually occur, there is an interest in utilizing these connections in order to enhance performance [20]. In this regard, graph neural networks (GNNs) have become increasingly popular, due to their ability to effectively process data within a graph structure [21]. As data privacy becomes essential, GNNs are expected to meet certain privacy concerns [22]. GNN techniques enhance FL training by leveraging connections within two types of graph information: graphs between clients and layer connectivity within deep neural networks [23]. By addressing variations in local model architectures among clients and by considering the neural network as a graph, GNNs offer a novel approach to balancing interclient variations [24]. For example, devices in close proximity experiencing similar conditions can be modeled as interclient graphs, where each device is represented by a node.

In the present study, we present DPS-GAT (differentially private selection-graph attention networks), a GNN-based architecture that jointly provides optimal resource allocation and client selection with DP guarantees in FL for healthcare. In particular, we solve the underlying optimization problem of client selection and resource allocation by implementing graph attention networks [25] and differential privacy, by extending our initial work [26]. For the purposes of our solution, we have achieved an optimal trade-off between client selection and resource allocation, while employing appropriate DP techniques. To the best of our knowledge, this is the first study that provides a GNN-based solution considering client selection, resource allocation, and DP in a DFL setting.

The paper is organized as follows: in Section 2 we present related work, while in Section 3 we proceed with our system model and the corresponding problem formulation. In Section 4 we present our contribution to the joint resource allocation and client selection with DP guarantees; in Section 5 we demonstrate the feasibility and performance of our architecture, and in Section 6 we provide the discussion of our results. Finally, in Section 7 we give our concluding remarks.

## 2. Related Work

### 2.1. Client Selection and Resource Allocation in Federated Learning

In [8], the authors highlight the significance of distributed learning in next-generation intelligent networks, emphasizing collaborative model training among intelligent agents without centralized raw data processing for privacy preservation. However, this highlights the challenge of high communication overhead in wireless systems with limited resources, prompting the need for communication-efficient distributed learning algorithms. Study [27] addresses resource efficiency in FL through intelligent participant selection and incorporating updates from straggling participants, resulting in improved model quality and optimized resource utilization, thus enhancing the effectiveness of the training process. In [28], the authors address the resource allocation problem in FL workflows where computational power, network bandwidth, and energy resources are limited. The study explores various resource allocation methods, considering factors such as node capabilities, dataset complexity, and FL workflow characteristics. The authors of [29] propose an innovative framework integrating edge computing with parallel split learning, to overcome challenges regarding privacy requirements on resource-constrained devices while reducing training latency and maintaining accuracy. In [30], the authors provide a comprehensive study of training FL algorithms in realistic wireless network scenarios. They solve the joint optimization problem involving learning, wireless resource allocation, and user selection. The authors of [31] propose FEDACS, a novel personalized FL algorithm that introduces an attention-based client selection mechanism, to mitigate challenges arising from non-IID data and data scarcity by prioritizing collaboration among clients with similar data distributions. The authors of [32] introduce a distributed cross-device FL framework, aiming to address limitations in existing centralized client selection approaches. In [33], the authors address the challenge of improving the learning performance of FL in over-the-air computation scenarios, particularly focusing on energy-harvesting clients. In [34], the authors introduce an FL framework augmented with critical learning periods (CLPs) [35]. By adaptively incorporating CLPs into existing FL methods, this significantly improves model accuracy while achieving better communication efficiency compared to the state-of-the-art methods.

### 2.2. Graph Neural Networks for Client Selection and Resource Allocation

The authors of [36] address the distributed power allocation problem in interference-limited wireless networks with dense transceiver pairs, through the design of low signaling overhead schemes using GNNs. In [37], the authors introduce a GNN designed to enhance bandwidth allocation for multiple legitimate wireless users transmitting to a base station in the presence of an eavesdropper. Furthermore, the authors of [38] introduce a GNN-based approach for DFL in D2D wireless networks, aiming to minimize total training delay and enhance learning performance. The proposed method employs a multi-head graph attention mechanism to capture diverse client and channel features, and it incorporates a neighbor selection module for clients to choose participating neighbors in model aggregation. To the same extent, the study of [39] proposes a solution to the challenges of dynamic bandwidth allocation in wireless vehicular networks, addressing mobility and heterogeneity with the first algorithm employing GNNs to predict vehicular network connection topology, prioritizing vehicles for bandwidth allocation based on quality of service (QoS) requirements and proximity. The authors of [40] introduce a novel graph-based client selection framework tailored for the heterogeneity in FL settings. This addresses the challenge of diverse hardware configurations and data distributions among mobile devices.

### 2.3. Differential Privacy in Federated Learning

DP deployment in FL environments has already been documented in the recent literature. Specifically, the authors of [41] introduce a solution to privacy concerns arising in FL by incorporating the concept of user-level differential privacy. In [42], the authors address the challenge of minimizing FL training delay over wireless channels while considering imbalanced data distributions, privacy constraints, and transmission quality. Study [43] introduces an iterative DP algorithm for client selection in FL, addressing scenarios where clients coordinate with a central server to complete tasks while deciding to participate based on local computation and probabilistic intent. The algorithm does not rely on client-to-client information exchange, and it ensures near-optimal values to clients over long-term average participation with a certain differential privacy guarantee. Following a different approach, the authors of [44] present a novel DP-enhanced FL platform that treats privacy as a resource and addresses the challenge of accumulating privacy leakage in multiple FL jobs. In [45], the authors propose a unified FL framework that integrates both horizontal and vertical FL approaches. The framework aims to balance privacy preservation, model utility, and system efficiency, crucial for large-scale model training and deployment. The paper formulates and quantifies trade-offs between privacy leakage, utility loss, and efficiency reduction.

## 3. System Model

We consider a D2D wireless network with *N* clients in a given area. The DFL process iterates for *R* training rounds. Considering the potential mobility of the clients, we model the network as a time-varying graph, namely $G_{r}\left(V,E_{r}\right)$, where *V* and $E_{r}$ denote the set of clients (nodes) and the set of communication links (edges) between nodes in the *r*-th training round, with r=1,2,⋯,R, respectively. Furthermore, we assume that there exists a communication link between two clients when their signal-to-interference-plus-noise ratio (SINR) is greater than or equal to a certain threshold, denoted by Ω [38]. We define the adjacency matrix in the *r*-th training round, denoted by $A^{r}$, in the following manner:

(1) $$A^{r}=\left(a_{i,j}^{r}\right),\;\mathrm{with}\;a_{i,j}^{r}\in \left\{0,1\right\},\;$$

where $a_{i,j}^{r}$ represents the link (or absence thereof) between client *i* and client *j*. The set of neighboring nodes of client *i* in the *r*-th training round is represented by $N_{i}^{r}$. Each client has a local dataset, $D_{i}$, with $D_{i}$ representing its data samples. Let parameters $\theta_{i}^{R}\in R^{d}$ denote the local model of client *i* after *R* training rounds. The loss function associated with the local model of client *i*, denoted by $L_{i}$, is defined as in [38], namely,

(2) $$L_{i}\left(\theta_{i}^{R}\right)=\frac{1}{D_{i}}\sum_{z\in D_{i}}\ell \left(z;\theta_{i}^{R}\right),\;$$

where $\ell (z;\theta_{i}^{R})$ denotes the loss on data sample $z\in D_{i}$ with model $\theta_{i}^{R}$.

Furthermore, in the *r*-th training round, client *i* trains its model locally with *K* stochastic gradient descent (SGD) steps. As in [37], we define client *i*’s model at the κ-th step as $\theta_{i}^{r,\kappa }$, 1⩽κ⩽K, updated as follows:

(3) $$\theta_{i}^{r,\kappa }=\theta_{i}^{r,\kappa -1}-\eta^{r}\nabla L_{i}\left(\theta_{i}^{r,\kappa -1}\right),\;$$

where $\eta^{r}$ and $\nabla L_{i}\left(\theta_{i}^{r,\kappa -1}\right)$ denote the learning rate and the gradient of the loss function of client *i* at the (κ−1)-th SGD step in the *r*-th training round, respectively.

In this work, each client selects a subset of its neighboring client devices, to participate in model aggregation. We define the neighbor selection decision as $s_{i,j}^{r}\in \left\{0,1\right\}$, satisfying

(4) $$s_{i,j}^{r}⩽a_{i,j}^{r},\;\forall \;i,j\in V.\;$$

The neighbor selection matrix for aggregation in the *r*-th training round is represented by $S^{r}\in R^{NxN}$, with $s_{i}^{r}\in R^{1xN}$ denoting the neighbor selection row vector of client *i*. We define the set of neighbors that client *i* has selected for aggregation in the *r*-th training round as ${\hat{N}}_{i}^{r}$, which includes the non-zero elements in vector $s_{i}^{r}$. Client *i* updates its local model by aggregating the local models of its selected neighbors in the *r*-th training round, as follows:

(5) $$\theta_{i}^{r+1}\left(s_{i}^{r}\right)=\sum_{j\in {\hat{N}}_{i}^{r}}p_{j,i}^{r}\theta_{j}^{r,K},\;p_{j,i}^{r}=\frac{D_{j}}{\sum_{n\in {\hat{N}}_{i}^{r}}D_{n}},\;$$

where $p_{j,i}^{r}$ represents the weight of the model of client *j* when client *i* performs aggregation at the end of the *r*-th training round. We note that $\theta_{i}^{r+1}$ will then be used as client *i*’s model for local training in the (r+1)-th training round.

In DFL, each client selects its neighbors, CPU frequency, and transmit power. The chosen values result in computation and communication delay. The computation delay of a client is the time that the client uses for local training. We denote the CPU frequency of client *i* with the variable $f_{i}^{r}\in \left[f_{i}^{min},f_{i}^{max}\right]$, where $f_{i}^{min}$ and $f_{i}^{max}$ represent the minimum and the maximum CPU frequency of client *i*, respectively. Denoting the number of CPU cycles of client *i* to process one bit of data by $c_{i}$, we define the computational delay as

(6) $$T_{i}^{c,r}=\frac{c_{i}D_{i}K}{f_{i}^{r}}.\;$$

In terms of communication delays, we define the transmit power of client *i* in the *r*-th training round as $p_{i}^{r}\in \left[0,p_{i}^{max}\right]$, with $p_{i}^{max}$ denoting the maximum transmit power of client *i*. Let $g_{i,j}^{r}$ denote the channel gain from client *i* to client *j* in the *r*-th training round. At time *t*, we define the set of clients that transmit their updated models as M(t)⊂V. The corresponding achievable data rate at time *t* in the *r*-th training round is denoted by $R_{i,j}^{r}\left(t\right)$ and is defined as [42]

(7) $$R_{i,j}^{r}\left(t\right)=B\log_{2}\left\{1+\frac{p_{i}^{r}g_{i,j}^{r}\left(t\right)}{\sum_{\kappa \in M(t)}{\left|g_{\kappa ,j}^{r}\right|}^{2}p_{\kappa }^{r}+\sigma^{2}}\right\},\;$$

where σ is the received noise power. Consequently, if we denote the communication delay by $T_{j,i}^{t,r}$, then, considering the different transmit power and channel gains of the selected clients and the changing inference over time, the delay is such that

(8) $$\int_{t^{r}}^{t^{r}+T_{j,i}^{t,r}}R_{i,j}^{r}\left(t\right)dt=\xi_{m},\;$$

where $t^{r}$ denotes the time that client *i* receives the model from client *j*, and where $\xi_{m}$ denotes the model size. Finally, $T_{i}^{t,r}=\max_{j}T_{j,i}^{t,r}$ represents the total time client *i* needs to receive the models from its selected neighbors. The total delay of the *r*-th training round is then expressed as the sum of the maximum values of communication and computation delays that are functions of the neighbor selection matrix, transmit power, and CPU frequency of all the clients, i.e.,

(9) $$T^{r}=\max_{i\in V}\left\{T_{i}^{c,r}+T_{i}^{t,r}\right\}.$$

Due to time-varying topologies, the neighbor selection and resource allocation decisions are independent across the training rounds. Here, we define as $d_{max}$ the maximal time interval of each communication round, which is used to avoid an endless waiting time caused by possible stragglers. The time that clients with available channels require in order to jointly complete an update of their respective FL models at the *r*-th communication round is given by

(10) $$d\left(r\right)=\max_{i\in V}\left\{\min\left\{T^{r},d_{max}\right\}\right\}.\;$$

In order to satisfy the underlying privacy requirements of DFL, we consider a DP mechanism with parameters ϵ and δ. In our study, the DP budget ϵ>0 is a bound on all outputs on neighboring datasets, namely $D_{i}$ and $D_{j}$, while δ represents the probability of the event that the ratio of the probabilities for the two adjacent datasets cannot be bounded by $e^{\epsilon }$ after adding a privacy-preserving mechanism. We employ local differential privacy (LDP), as in [46], by applying perturbation mechanisms on the user’s datasets. We adopt the Gaussian mechanism that has been widely used in privacy-preserving SGD algorithms: this mechanism satisfies LDP for the *r*-th client, when we properly select the value of the standard deviation (STD), $\sigma_{i}$, for each client [41]. As in [47], the selected composition of leakage, ${\hat{\epsilon }}_{i}$, based on local privacy leakage, is given by

(11) $${\hat{\epsilon }}_{i}=\sqrt{\frac{E_{i}\ln\left(\frac{1}{\delta_{i}}\right)}{\ln\left(\frac{2}{\delta_{i}}\right)}}\epsilon_{i},$$

where $E_{i}$ is the number of model uploads of the *r*-th client. Thus, it is crucial to control the largest possible delay among all the clients. Having defined the system model, the next step is to design the dynamic channel assigning mechanisms in this work, to minimize time delay while completing the training. Following the approach of [42], the problem is formulated as follows:

(12a) $$\min_{s_{i}^{r}\in S^{r}}\sum_{r\in R}d\left(r\right),\;$$

subject to

(12b) $$s_{i,j}^{r}⩽a_{i,j}^{r},\;i,j\in V,\;$$

(12c) $$0⩽p_{i}^{r}⩽p_{i}^{max},\;i\in V,\;$$

(12d) $$f_{i}^{min}⩽f_{i}^{r}⩽f_{i}^{max},\;i\in V,\;$$

(12e) $$\lim_{r\in R}\sup\frac{1}{R}\sum_{r}I_{i,j}\left(r\right)⩾\beta_{i},\;$$

where $\beta_{i}$ denotes the participating ratio for client *i*, determined by its DP requirement $\left(\epsilon_{i},\delta_{i}\right)$ and local training model, and $I_{i,j}\left(r\right)$ denotes an indicator function, i.e., whether the local training model of client *j* is successfully received by client *i* at the *r*-th training round. Equation (12a) implies that the time used for the update of the local models is determined by the neighboring selection matrix $s_{i,j}^{r}$. It is worth mentioning that even when a minimum positive $\epsilon_{i}$ is considered there are different responses in the local model training of the clients, and, as a consequence, their potential selection is affected [42]. In practice, the local training models for imaging classification tasks can be deployed across medical institutions and devices participating in the FL process. The computational requirements primarily depend on the complexity of the selected neural network models and the volume of data processed by each client. Typically, clients require moderate computational capabilities, including multi-core CPUs and GPUs, to handle local model training. The DPS-GAT model can be trained offline, considering periodic retraining when there are new complex graph structures and considerable scalability differences.

## 4. Graph Neural Networks for Differentially Private Client Selection and Resource Allocation

**Figure 2:**
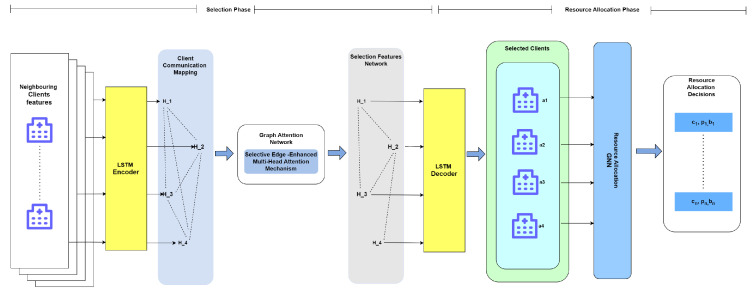
DPS-GAT approach.

For the purposes of our study, we propose a multi-head graph attention network (GAT) [25]. The attention mechanism can help each client learn the features of its neighboring clients and determine their importance score for selection. The DPS-GAT algorithm consists of an encoder, a selection features network, and a decoder (see Figure 2). In order to capture the intrinsic properties of D2D networks, the encoder encodes client features, including the maximum transmit power, the maximum and minimum CPU frequencies, the differential privacy budget, and the edge features that represent channel gains. The neighboring selection network determines the set of neighboring clients for model aggregation. The decoder determines the final allocation decisions of the selected clients. Let

(13) $$x_{i}^{f,r}=\left(p_{i}^{max},f_{i}^{min},f_{i}^{max},\epsilon_{i}^{min}\right),$$

denote the feature vector of client *i*. Similarly, the edge feature, denoted by $x^{e,r}$, represents the absolute value of the channel gain of link (i,j).

### 4.1. Encoder

We consider both node and edge features. Node features represent the communication and computation capabilities of devices. Edge features characterize the channel gain affecting the communication delay. The client encoder utilizes these two features, in order to capture the intrinsic properties of the network topology. We employ the *z*-score normalization to stabilize the gradients of GNN [48]. Since each client aggregates features of different magnitudes from its neighbors, local normalization is applied, to ensure that the mean and standard deviation of the features are zero and one, respectively [38]. In this work, we deploy long short-term memory (LSTM) as the encoder for the features of the clients. The features of client *i* at the training round *r*, denoted by $r_{r}^{\left(i\right)}$, are defined as

(14) $$r_{r}^{\left(i\right)}=\mathrm{LSTM}\left(x_{i}^{f,r}\right).$$

The features of all clients will then be gathered as a set, denoted by $R_{r}$, namely,

(15) $$R_{r}=\left\{r_{r}^{\left(i\right)},\cdots ,r_{r}^{\left(N\right)}\right\}.\;$$

Furthermore, we denote by $S_{r}$ the selection decisions of the clients, i.e.,

(16) $$S_{r}=\left\{s_{r}^{\left(0\right)},s_{r}^{\left(1\right)},...,s_{r}^{\left(N\right)}\right\},\;$$

where $s_{r}^{\left(i\right)}$ represents the selection feature of agent *i* at the training round *r*. Our GAT-based algorithm models the features of all clients simultaneously, in the sense that

(17) $$S_{r}=\mathrm{GAT}\left(R_{r},E_{r}\right),\;$$

where $E_{r}$ represents the set that contains the edge features at the training round *r*.

### 4.2. Selection Features Network

We proceed with the process of neighboring selection by considering the client features $R_{r}$, defined in Equation (15). The selection representation occurs via a GNN that handles all nodes, edges, and continuous edge features. By aggregating information from neighbors, several GAT layers are employed, with each layer updating node features, denoted by $h_{i}$, i=1,2,⋯,N. A layer first transforms node and edge features accordingly, and then it aggregates neighboring node features with a multi-head attention mechanism. This combination allows GAT layers to capture dependencies between nodes. GAT provides updated node features based on the information received by its neighbor. A concatenated feature vector, $e_{ij}^{r}$, defined by

(18) $$e_{ij}^{r}=\left[e_{ij}\right]\left[h_{i}\right]\left[h_{j}\right],$$

represents the edge feature of node *j* from node *i*’s point of view, which is then forwarded to a shared attention mechanism. The attention mechanism, $a^{T}$, is a single-layer, feed-forward neural network with LeakyRelu, softmax linearization, and nonlinearities, such as the sigmoid. Its attention coefficients, $a_{ij}$, indicate the importance of node *j* to node *i*. Subsequently, we compute the node attention scores, in order to generate the node embeddings, namely,

(19) $$a_{ij}=\frac{\exp\left(\mathrm{LeakyReLU}\left(a^{T}\left[h_{i}\right]\left[e_{ij}^{r}\right]\right)\right)}{\sum_{k\in {\hat{N}}_{i}}\exp\left(\mathrm{LeakyReLU}\left(a^{T}\left[h_{i}\right]\left[e_{ik}^{r}\right]\right)\right)}.\;$$

We implement independent attention mechanisms per node. Specifically, the multi-head attention mechanism is shaped by *K* independent attention mechanisms, with their features to be concatenated, resulting in the following output feature representation:

(20) $$\;h_{i}^{new}=\sigma \left(\sum_{j\in {\hat{N}}_{i}}\left[a_{ij}^{1}W_{h}^{1}e_{ij}^{r}\right]\left[a_{ij}^{2}W_{h}^{2}e_{ij}^{r}\right]\cdots \left[a_{ij}^{K}W_{h}^{K}e_{ij}^{r}\right]\right),$$

with σ(·), $W_{h}$, and *k* representing the sigmoid function, the weighted sum of node features over its neighborhood, and the number of attention heads, respectively. When performing training in DFL, the full participation scheme may incur high communication delays. Thus, we propose a neighbor selection module to determine which neighbors are selected for each client. The inputs to the module consist of the concatenated embedding of a client and the embeddings of its neighbors. The concatenated embedding of client *i* and its neighbor *j* is $h_{i,j}^{new,r}=\left[h_{i}^{new,r}\right]\left[h_{j}^{new,r}\right]$. We apply a decoder for the neighboring selection decisions and the resource allocation matrix. For each neighboring client $j\in {\hat{N}}_{i}^{r}$, client *i* determines the selection decision $s_{i,j}$ based on a predefined threshold γ∈(0,1). Since bi-directional model exchanges are required for model aggregation in DFL, we require

(21) $$e_{i,j}^{r}⩾\gamma ,\;e_{j,i}^{r}⩾\gamma .$$

### 4.3. Decoder

We deploy an LSTM decoder as

(22) $$f_{r}^{\left(i\right)}=\mathrm{LSTM}\left(\left[r_{r}^{\left(i\right)}\right]\left[s_{r}^{\left(i\right)}\right]\right),\;$$

with $\left[r_{r}^{\left(i\right)}\right]\left[s_{r}^{\left(i\right)}\right]$ representing the concatenation of the corresponding features and $f_{r}$ denoting the selection features of the nodes. The outcome includes only the features of the selected nodes for resource allocation. We further employ a full GNN block containing a global block, a node block, and an edge block. Adopting a set-to-set mapping approach similar to the one in [49], we define the resource allocation GNN, denoted by RAG, as

(23) $$\mathrm{RAG}=\left\{Q_{enc}^{u},Q_{enc}^{e},Q_{dec}^{u}\right\},$$

where we adopt the following notation: $Q_{enc}^{u}$: $R^{2}\to R^{n_{u}}$ represents the encoding rates of the node features (transmit power, CPU frequencies, privacy constraints), $Q_{enc}^{e}$: $R^{2}\to R^{n_{e}}$ the encoding rates of the channel gains between the clients, $Q_{dec}^{u}$: $R^{n_{u}}\to R$ the decoding of the final state for the assignment of resources, and $\left(Q^{e},Q^{u},Q^{b}\right)$: $R^{n_{u}+n_{e}+n_{b}}\to R^{n_{e}}$, $R^{n_{u}}$ or $R^{n_{b}}$ represent the updates of the graph’s edges, nodes, and global values, respectively. Furthermore, $n_{u}$, $n_{e}$, and $n_{b}$ denote the hyperparameters that represent the size of the node encodings, the edges, and the final resource assignments, respectively.

To ensure that the proposed algorithm can adapt to different network settings, we train our GNN in an offline and unsupervised manner. We generate a set of D2D network scenarios with topology changes and time-varying channel conditions. Due to the small size of the neighbors’ node features, compressed model parameters, and channel conditions, each client collects these features in each scenario with negligible communication delay. It then determines the decision and sends the decision to its neighbors, and the loss of each client can be calculated.

## 5. Results

For the evaluation of our model, we employed the Regensburg pediatric appendicitis dataset [50], the Breast Ultrasound Image dataset [51], and the Maternal-Fetal US [52] dataset. These datasets were used for image classification in clients. We considered four distinct scenarios with several differential privacy budgets $\left(\epsilon_{i}\right)$. We ensured that each client’s local dataset contained an equal number of data samples [53]. The clients were randomly positioned in an area following the uniform distribution. We assumed that each client, i.e., a certain hospital or medical institution, was stable. In our approach, we did not consider the distances between the clients, as the underlying transmit power, bandwidth allocation, and DP budgets completely defined the client selection process and the model weights exchange. In this regard, we considered the transit power of each device to follow a uniform distribution [8,15] mW. The frequency of each client device was set to $f_{i}^{min}\in \left[0.1,0.2\right]$ GHz and $f_{i}^{max}\in \left[1.5,2.5\right]$ GHz. To evaluate the trade-offs between training loss and privacy strength, the privacy budget values $\left(\epsilon_{i}\right)$ investigated were

- the same for every client with $\epsilon_{i}\in \left\{0.1,0.5,0.9\right\};$
- different among the client population with $\epsilon_{i}$ values in the interval (0.01,1), following a uniform distribution.

We initialized the transmit power and CPU frequency of each client to the maximum value and then we implemented our trained GNN. Each client could independently make their own decisions and determine the delay by recording the wall clock time. Our experiments were deployed on a Windows 11 Home HP AMD Ryzen 7 5825U, utilizing Python 3.8 and PyTorch libraries. Indicatively, for testing the model performance we considered 50 clients, while for the total delay we considered scenarios of between 5 and 50 clients. Furthermore, we adopted a supervised learning approach by implementing the framework of [54] within our DFL setting. In order to test how bandwidth affected both the model performance and the client selection process, we omitted any constraint distances among the clients, and the overall bandwidth quantity was set to 2000 Mbps.

**Figure 3:**
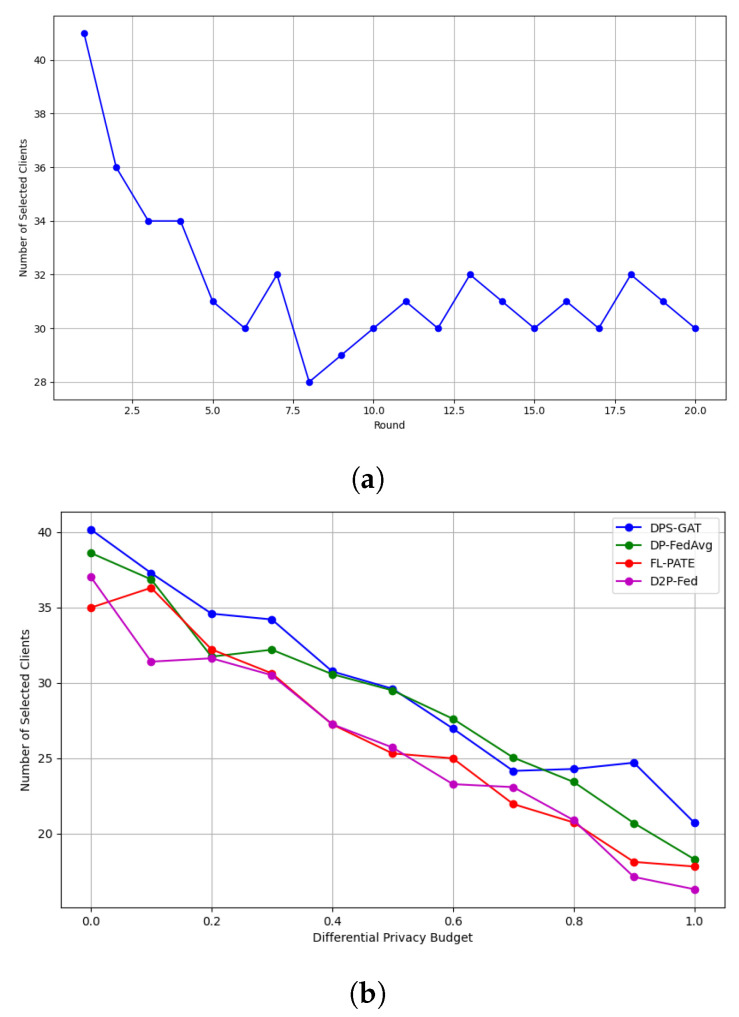
Number of selected clients. (**a**) Number of selected clients per communication round under DP heterogeneity. (**b**) Number of selected clients per DP budget in the first round.

**Figure 4:**
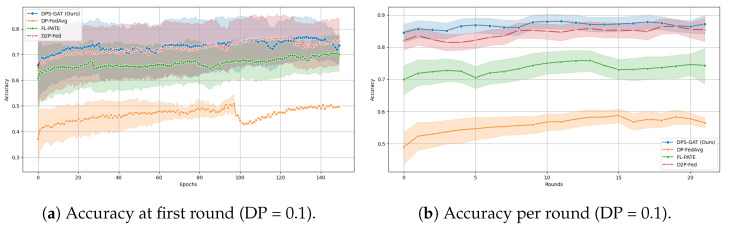

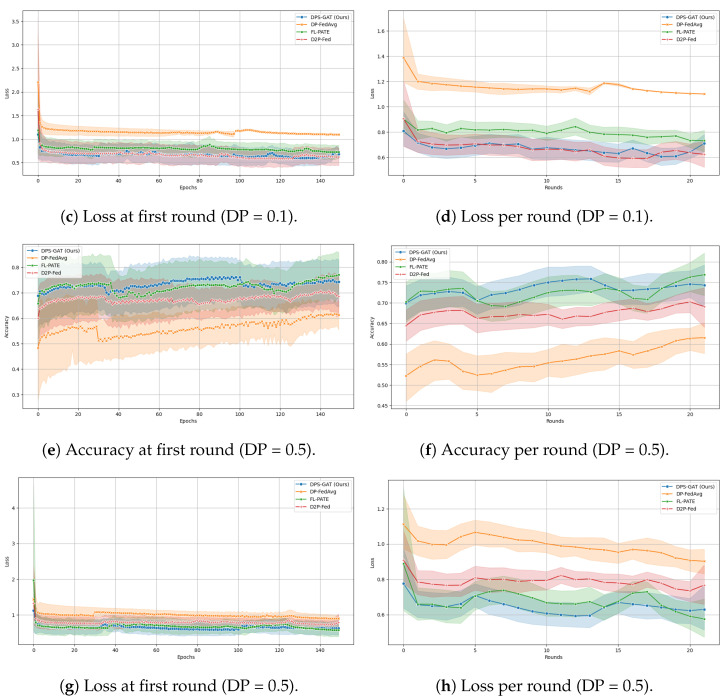
Comparison of accuracy and loss for DP budget values ϵ=0.1 and ϵ=0.5.

**Figure 5:**
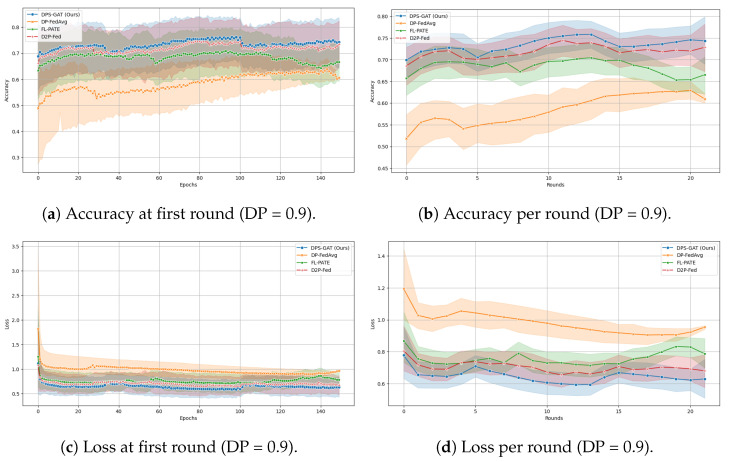

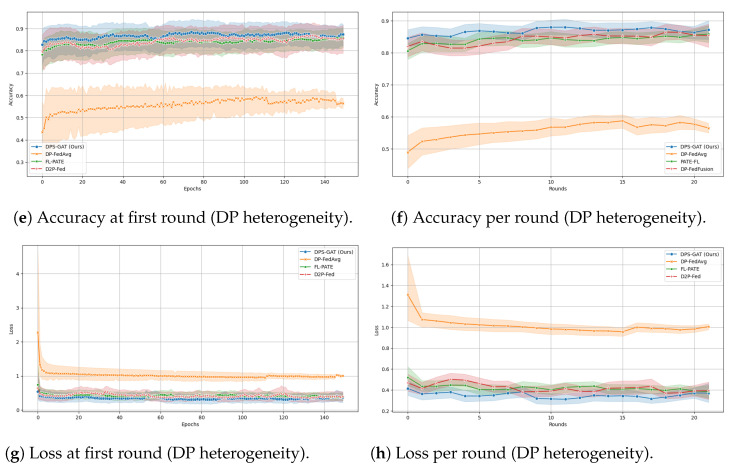
Comparison of accuracy and loss for a DP value ϵ = 0.9 and for DP budgets under heterogeneity.

In Figure 3a, we present the number of selected clients per communication round in the case of DP heterogeneity. On the other hand, in Figure 3b, we present the number of selected clients under the same ϵ values in the first communication round. To test and validate our proposed algorithm, we made performance comparisons (accuracy and loss computations) with three advanced differentially private client selection algorithms from the literature (see Figure 4 and Figure 5):

i. DP-FedAvg (differentially private federated averaging) [55] was selected, due to its foundational role in FL with differential privacy, serving as a standard benchmark for comparisons.
ii. FL-PATE (private aggregation of teacher ensembles for federated learning) [56] offers an innovative approach by ensuring privacy through aggregating teacher model outputs. This method differs from DP-FedAvg and is particularly suited for scenarios requiring stringent privacy guarantees.
iii. D2P-Fed [57] was chosen for its recent advancements, integrating advanced mechanisms for privacy-preserving client selection and update aggregation.

We present our results on the Breast Ultrasound Image dataset [51], and the Maternal-Fetal US [52] dataset, in terms of average performance per round, total delay, and number of selected clients per round in Table 1, Table 2 and Table 3, respectively.

**Figure 6:**
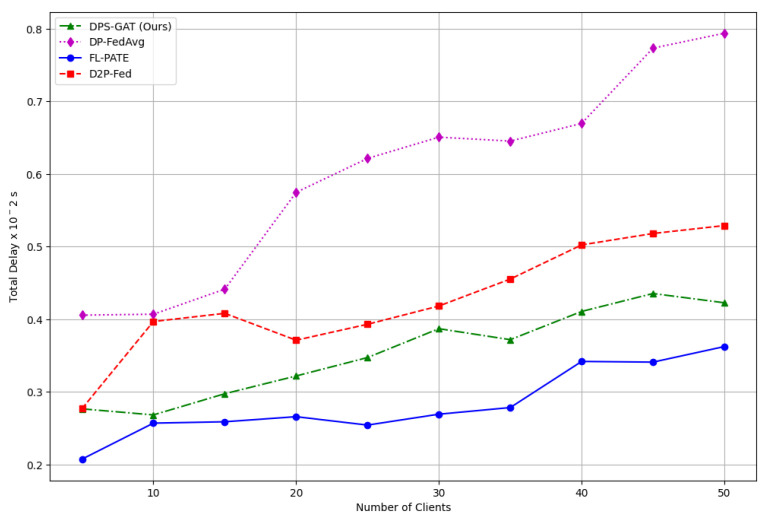
Total delay.

**Figure 7:**
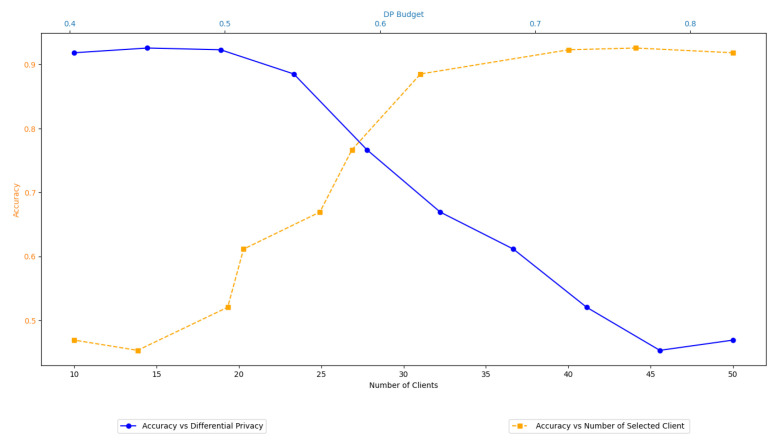
Learning–differential privacy–selected clients’ trade-offs.

Furthermore, we computed the total communication and computation delay for several numbers of participating clients with different DP budget values (see Figure 6), as well as the given trade-offs between learning performance, differential privacy, and client selection, as depicted in Figure 7.

## 6. Discussion

In this study, we investigated the performance of our proposed DPS-GAT algorithm in the context of client selection for FL, considering resource constraints with differential privacy guarantees in medical applications. Our goal was to assess the efficacy of DPS-GAT in selecting an optimal number of clients per training round while ensuring privacy preservation without violating the training performance. The evaluation of different DP budgets (spanning from 0.1 to 0.9) reveals that DPS-GAT effectively adjusts to diverse privacy needs. Given different ϵ values, the number of selected clients remains relatively stable, especially after the 15th communication round. This stability is crucial, as it guarantees consistent model performance and convergence throughout extended training periods, even as privacy constraints become more stringent.

**Figure 8:**
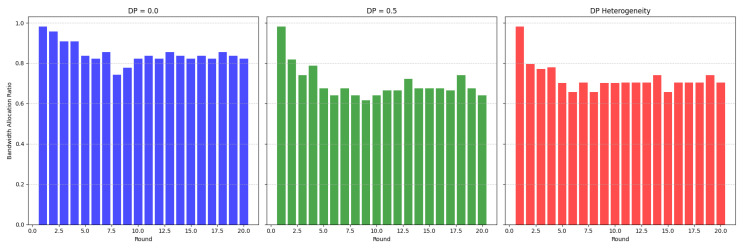
Bandwidth allocation ratio (round 1, N=41 clients).

In benchmarking terms and all three datasets, DPS-GAT reached and outperformed the baseline algorithms, in terms of training performance, in relation to the number of selected clients per communication round. DPS-GAT showed a stable client selection range as the ϵ value increased, maintaining a higher client selection rate compared to DP-FedAvg, FL-PATE, and D2P-Fed, while it also maintained a balance in the bandwidth allocation ratio per round, as depicted in Figure 8. DPS-GAT demonstrated high training accuracy compared to the other differentially private algorithms. Specifically, in the case of the Regensburg pediatric appendicitis dataset [50], it consistently achieved accuracy of over 0.7 in every DP budget scenario, reaching approximately 0.82 in cases of DP budget heterogeneity among clients. This trend persisted throughout all the communication rounds of our experiments. Furthermore, the accuracy trade-off between the number of selected clients and the DP budget indicated that there was a specific interval of reference within which to maintain high training accuracy, while not compromising the average DP budget. This balance also ensured that resource consumption remained low, with an optimal number of selected clients (*N* = 27 clients with an average ϵ = 0.6). In the total communication and computation delay for several numbers of participating clients, DPS-GAT was the second-best against its three counterpart algorithms in the ϵ heterogeneity case.

Compared to DP-FedAvg, FL-PATE, and D2P-Fed, which showed moderate fluctuations and a slightly lower client selection rate, DPS-GAT demonstrated superior stability and higher average client selection. This highlights the efficiency of our algorithm in managing privacy-preserving constraints while maximizing client participation. The ability of DPS-GAT to maintain a high and stable number of client selections across various rounds and DP budgets has significant practical implications. It indicates that FL systems can achieve strong privacy guarantees without compromising client engagement and model performance. This balance is essential for real-world applications where both privacy and performance are paramount. However, in practice, there are cases where the network experiences certain perturbations. In these cases, where loss of communication with a client occurs, our DPS-GAT algorithm excludes the client from the training round. If communication is interrupted during training, the updates from the affected client are excluded, and training proceeds with the remaining clients, maintaining model robustness. In this regard, small-world network topologies are ideal, due to their short path lengths and high clustering, enhancing resilience and communication efficiency [58]. Scale-free networks ensure robustness, with highly connected hub nodes that maintain connectivity despite node failures [59]. These topologies can collectively ensure efficient, robust, and scalable FL, making them ideal for our system.

The scalability of our DPS-GAT method is vital for DFL in medical applications. Utilizing GNNs for client selection and resource allocation ensures efficient handling of large, complex networks. Our differential privacy mechanisms scale with the number of clients, maintaining privacy and performance. The multi-head attention mechanism in GNNs allows parallel processing, enhancing scalability. Our experimental results confirm that DPS-GAT maintains high accuracy and low latency as the network size grows, making it ideal for large-scale healthcare deployments. Furthermore, our proposed DPS-GAT algorithm demonstrated a robust ability to manage the inherent trade-offs in FL with differential privacy. The results underscore its superiority over existing baseline algorithms, making it a promising solution for privacy-preserving FL-based medical applications.

Our research demonstrates that the DPS-GAT algorithm significantly improves the robustness and efficiency of DFL in medical applications by ensuring high model accuracy and strong privacy preservation. This advancement has the potential to enhance patient care through more accurate predictive models while maintaining stringent privacy standards, paving the way for broader adoption of FL in sensitive domains.

## 7. Conclusions

In this work, we present a novel and effective solution to the challenges of DFL for healthcare applications. By introducing a GNN-based approach, our study simultaneously addresses efficient client selection and resource allocation with DP guarantees. Our results highlight the importance of incorporating privacy-preserving techniques in FL for healthcare. Furthermore, GNN methods demonstrate a highly promising direction for considering privacy requirements in modern DFL environments without compromising the underlying training performance. Future work could explore the scalability of DPS-GAT in more diverse and larger FL environments, particularly within medical settings where data diversity and volume are substantial.

## Figures and Tables

**Table 1:** Performance of DPS-GAT for the datasets [51,52].

Datasets	Algorithms	Average Performance per Round (20 Rounds)			
		DP = 0.1	DP = 0.5	DP = 0.9	DP Heterogeneity
		Accuracy (Loss)	Accuracy (Loss)	Accuracy (Loss)	Accuracy (Loss)
[51]	DPS-GAT	0.821 (0.219)	0.826 (0.217)	0.828 (0.216)	0.834 (0.210)
	DP-FedAvg	0.817 (0.231)	0.822 (0.225)	0.824 (0.224)	0.829 (1.112)
	FL-PATE	0.812 (0.220)	0.819 (0.223)	0.821 (0.222)	0.816 (0.245)
	D2P-Fed	0.805 (0.214)	0.816 (0.210)	0.818 (0.209)	0.807 (0.187)
[52]	DPS-GAT	0.810 (0.122)	0.812 (0.111)	0.814 (0.113)	0.816 (0.116)
	DP-FedAvg	0.805 (0.129)	0.807 (0.128)	0.809 (0.127)	0.802 (1.254)
	FL-PATE	0.800 (0.125)	0.802 (0.126)	0.804 (0.125)	0.795 (0.238)
	D2P-Fed	0.795 (0.117)	0.797 (0.113)	0.799 (0.112)	0.809 (0.139)

**Table 2:** Total delay per algorithm for the datasets [51,52] versus client number.

Datasets	Algorithms	Total Delay (Seconds)				
		10 Clients	20 Clients	30 Clients	40 Clients	50 Clients
[51]	DPS-GAT	0.243	0.291	0.344	0.390	0.447
	DP-FedAvg	0.752	0.807	0.855	0.864	0.872
	FL-PATE	0.220	0.273	0.329	0.378	0.421
	D2P-Fed	0.236	0.284	0.332	0.385	0.431
[52]	DPS-GAT	0.245	0.292	0.346	0.397	0.444
	DP-FedAvg	0.759	0.809	0.851	0.862	0.876
	FL-PATE	0.227	0.274	0.325	0.377	0.423
	D2P-Fed	0.235	0.285	0.334	0.388	0.430

**Table 3:** Total number of client selection per algorithm for the datasets [51,52].

Datasets	Algorithms	Communication Rounds			
		5th	10th	15th	20th
[51]	DPS-GAT	12	15	17	20
	DP-FedAvg	38	40	43	43
	FL-PATE	11	14	16	19
	D2P-Fed	12	14	17	19
[52]	DPS-GAT	12	15	17	20
	DP-FedAvg	38	40	43	43
	FL-PATE	11	14	16	19
	D2P-Fed	12	14	17	19

## Data Availability

The data used for the experiments of this study are available from the Regensburg pediatric appendicitis dataset [50], the Breast Ultrasound Image dataset [51], and the Maternal-Fetal US dataset [52], which are open datasets.
